# Upgrading Behavioral Models for the Design of Digital Predistorters

**DOI:** 10.3390/s21165350

**Published:** 2021-08-08

**Authors:** Carlos Crespo-Cadenas, María José Madero-Ayora, Juan A. Becerra

**Affiliations:** Departamento de Teoría de la Señal y Comunicaciones, Escuela Técnica Superior de Ingeniería, Universidad de Sevilla, Camino de los Descubrimientos, s/n, 41092 Seville, Spain; ccrespo@us.es (C.C.-C.); jabecerra@us.es (J.A.B.)

**Keywords:** behavioral modeling, digital predistortion, nonlinear model identification, power amplifier linearization, Volterra series

## Abstract

This work presents a strategy to upgrade models for power amplifier (PA) behavioral modeling and digital predistortion (DPD). These incomplete structures are the consequence of nonlinear order and memory depth model truncation with the purpose of reducing the demand of the limited computational resources available in standard processors. On the other hand, the alternative use of model structures pruned a priori does not guarantee that every significant term is included. To improve the limited performance of an incomplete model, a general procedure to augment its structure by incorporating significant terms is demonstrated. The sparse nature of the problem allows a successive search incorporating additional terms with higher nonlinear order and memory depth. This approach is investigated in the modeling and linearization of a commercial class AB PA operating at a compression point of about 6 dB, and a class J PA operating near saturation. Results highlight the capabilities of this upgrading procedure in the improvement of linearization capabilities of DPDs.

## 1. Introduction

The deployment of modern wireless communication systems, based on spectrally efficient modulation schemes such as the orthogonal frequency division multiplexing (OFDM), is perhaps the main agent that has pushed the dawn of new ultra-linear and highly efficient power amplifiers (PAs) [1]. Advanced techniques in the discrete-time domain have been employed to quantitatively mitigate the nonlinear impairments generated by the PA and the IQ modulator, mainly through digital predistorters (DPD) in transmitters linearization [2,3,4,5], and also by applying post-compensation in the communications receiver [6,7].

The success of baseband signal processing techniques comes on a base of two aspects. First, the implementation of improved black-box models allowing superior accuracy and a better representation of the transmitter and receiver nonlinearities. Both neural networks and Volterra nonlinear filters have been advanced for the associated signal processing [8,9,10], but perhaps the most widely used approach in PAs linearization is the baseband Volterra model [11], whose discrete-time version, truncated in nonlinear order and memory length, is denoted as the full Volterra (FV) model. Regrettably, the size of its regressor set can be unsuitably large, and ad hoc models with a reduced set of regressors were proposed. For example, the memory polynomial (MP) model and its modifications [12,13], together with the generalized memory polynomial (GMP) [2] model, have demonstrated satisfactory performance in the design of a DPD. Another approach based on available information at the circuit level has also made possible the deduction of a reduced-order structure that contains the GMP lagging envelope terms with even-order envelope powers as a particular regressor type [14].

On the other hand, the adoption of compressed sensing (CS) techniques for the search of active regressors is a way to reduce the order in sparse systems [9,15,16,17,18] and has been particularized to PA Volterra models [19]. Likewise, the complete structure of the popular GMP model can be sparse, so that pruning methods have been applied to discard unimportant terms and reduce the regressor set [20,21,22]. The selection of the nonlinear orders and memory depths is performed by combining a greedy algorithm, the orthogonal matching pursuit (OMP) in [19] or the doubly orthogonal matching pursuit (DOMP) in [21], and a Bayesian information criterion (BIC) for determining the optimum number of coefficients. The proposal in [22] is based on a hill-climbing (HC) algorithm, which provides the best trade-off between modeling accuracy and model complexity by searching in the GMP structure.

Notwithstanding the notable performance of these approaches, a general concern exists on techniques to upgrade and optimize a given model. This interest has motivated recent publications which compare model pruning or model growing techniques [23,24]. In [24], the model growth is made taking into account only the initial set of the GMP regressors, but it could be necessary the upgrade with regressors not included in the complete GMP model. Unfortunately, no results have been published for an HC algorithm applied to a general FV model because the regressor set size is unsuitably large. Its massive number of regressors can also limit the use of matching pursuits [19,21] and the selection of a manageable model with reduced nonlinear order and memory depth may lead to a selected subset with a lack of significant terms.

The cited works evidence the need for a procedure to upgrade sub-optimal models in multiple scenarios. In the first one, which can be denoted as *intra-model* scenario, the search of new active regressors is circumscribed to the same model, e.g., the GMP in [22,23,24]. In a second *inter-model* scenario, the search is extended to a new model with a richer set of regressors so that the optimal model will benefit from a boost provided by the regressors of the second model. Several situations can be foreseen in this inter-model scenario: a memoryless (ML) model enhanced with memory regressors, a GMP model enhanced with FV regressors, or even an FV model enhanced with image signal regressors of the complex-valued Volterra series (CVS) model [25] when modulator impairments are significant, to mention only a few examples.

This communication formulates the proposal of an algorithm to upgrade a sub-optimal PA baseband model following a regressors pursuit procedure based on compressed sensing and the BIC rule. This approach is valid in both scenarios and is first illustrated with a sub-optimal pruned model resulting from a standard search applied to an FV model with a manageable, though incomplete, raw set of regressors. The smaller size of this pruned subset allows its enrichment with regressors that extend the nonlinear order or the memory depth, and a second search is performed. The result is a model with a small subset of significant regressors, including those with high nonlinear orders and large memory, that avoids computational constraints. In a second example, a ML sub-optimal model is improved with memory regressors. In Section 2, the perspective of enhancing a sub-optimal model is theoretically established, and modeling results for practical PAs are presented. Section 3 is devoted to the design of DPDs following the proposed upgrading procedure and its performance improvement is demonstrated. Finally, some concluding remarks are presented in Section 4.

## 2. A Strategy to Upgrade PA Models

The complex envelope at the output of a PA can be described with a discrete-time version of the baseband Volterra model advanced in [11]. In that case, the amplifier output is described by a linear combination of basis functions given by monomials resulting from the multiplication of delayed samples of the input complex envelope x(k) and its conjugate $x^{*}\left(k\right)$. If the series of these Volterra regressors is truncated to a maximum nonlinear order, the resulting structure is denoted as the FV model.

A severe drawback of the FV model is that its regressors stock is too large, involving a high computational cost in its regression and output signal generation. Thus, the identification of a pruned set of regressors is the main objective of model-order reduction techniques. The structures in [2,14] are examples of pruned models with reduced sets of regressors. Alternatively, it is possible to search within the whole FV-regressors set those active regressors that guarantee the FV performance.

Gathering $N_{s}$ samples of the PA input signal to arrange the column vector x, and defining similarly the Volterra regressor vectors $\xi_{i}$ arranged in an ordered fashion, the FV model can be expressed in matrix form as

(1) $$y=\sum_{i=1}^{N_{R}}h_{i}\xi_{i}=Xh,$$

where y is the column vector containing PA output samples, $N_{R}$ is the number of regressors, $h_{i}$ are the regression coefficients, $X=[\xi_{1}\;\xi_{2}\;\cdots \;\xi_{N_{R}}]$ is the regressors matrix and h is a column vector with the coefficients $h_{i}$. It is convenient to normalize the regressors in power, so the columns of X are taken to be unit-norm. As for a given nonlinear order and memory length, X contains the complete set of regressors of the FV model (1), here and below we refer to X as the whole FV-regressors matrix. The coefficients vector of (1) can be estimated with the standard least-squares (LS) algorithm

(2) $$\hat{h}={\left(X^{H}X\right)}^{-1}X^{H}y.$$

In a realistic scenario, it is possible to exploit the sparsity of the system to reduce the whole FV-regressors set by identifying only a small portion of active regressors. A suitable pruning procedure is [19,21]

- the application of a greedy pursuit for the search of the most significant regressors among the whole set of the FV model, and
- a criterion, such as the BIC, to stop the algorithm execution and avoid overfitting.

However, if the number of regressors is so large that the search step exceeds the computational resources, it is necessary to select a lower nonlinear order or memory to reduce the number of regressors in (1). After determining the best-reduced model, the regressors matrix of the sparse model $X_{a}=\left[\xi_{1}\;\xi_{2}\;\cdots \;\xi_{S}\right]$ is assembled from the matrix of an FV model with a shortfall in the regressors stock, with all but the *S* selected regressors set to zero ($S\ll N_{R}$). On the other hand, if the procedure is applied to the GMP, or any other a priori pruned model, we cannot affirm that the identified set is complete because the richness of the initial set of regressors may be insufficient and there is no guarantee that the selected set of active regressors achieves the best performance. Therefore, a method to complete the model structure and improve its performance would also be beneficial.

The present upgrading procedure is applied to a model with a deficit of active regressors. Assuming that the matrix $X_{a}$ of this incomplete model is known, the output signal predicted with the estimated parameters is

(3) $${\hat{y}}_{a}=X_{a}{\hat{h}}_{a},$$

and the residual vector is

(4) $$r^{\left(S\right)}=y-X_{a}{\hat{h}}_{a}.$$

The procedure proposed in this paper is to upgrade the incomplete model starting with the attachment of new stock of FV normalized regressors with higher nonlinear order and/or memory depth. The matrix of the additional model $X_{b}=\left[\xi_{S+1}\;\xi_{S+2}\;\cdots \;\right]$ is constructed with new regressors and is attached to $X_{a}$, thus forming the extended matrix $X=[X_{a}\;X_{b}]$. By way of illustration, if the pruned matrix $X_{a}$ was determined from a (2p+1) th-order FV model, a possible extension is $X_{b}$ constituted only by higher-order regressors.

The search of the supplementary active regressors starts with the definition of the first auxiliary basis function $\phi_{1}^{\left(1\right)}=\xi_{1}$. Following a procedure based on the Gram-Schmidt algorithm [20,21], the other Volterra regressors $\xi_{i}$, i=2,3,… are orthogonally projected onto the line $\phi_{1}^{\left(1\right)}$ spans, and the projections are subtracted to the original basis yielding the basis functions orthogonal to $\xi_{1}$,

(5) $$\phi_{i}^{\left(1\right)}=\xi_{i}-\frac{\xi_{1}^{H}\xi_{i}}{{\left|\xi_{1}\right|}^{2}}\xi_{1}.$$

Repeating the procedure for the remaining $\xi_{j}$ (j=2,…,S), the FV regressors of the additional model $X_{b}$ are transformed to a new set of basis functions $Z_{b}=\left[\phi_{S+1}^{\left(S\right)},\phi_{S+2}^{\left(S\right)}\cdots \right]$ orthogonal to the FV regressors $\xi_{i}$ of the incomplete model $X_{a}$. The extended auxiliary matrix is

(6) $$Z^{\left(S\right)}=\left[Z_{a}\;Z_{b}\right]=\left[\phi_{1}^{\left(S\right)}\;\cdots \phi_{S}^{\left(S\right)}\;\phi_{S+1}^{\left(S\right)}\;\phi_{S+2}^{\left(S\right)}\;\cdots \right].$$

The matrix $Z_{a}$ can be considered the result for the search of the first *S* active regressors at iteration t=S, and the residual vector can be calculated with (4). Continuing the search, at iteration t=S+1, $r^{\left(S\right)}$ is expressed as a linear combination of the new set of regressors. Then, the algorithm chooses the regressor of the additional $Z_{b}$ that better predicts the residual at iteration *t*, obtained by maximizing the absolute value of the projection of the regressors with the residual of the previous iteration

(7) $$i^{\left(t\right)}=\arg\max_{i\notin S^{\left(t-1\right)}}\left|\frac{Z_{\left\{i\right\}}^{(t)H}r^{\left(t-1\right)}}{∥Z_{\left\{i\right\}}^{(t)H}{∥}_{2}}\right|.$$

The chosen index is incorporated into the support set of the active coefficients and a new estimation of the coefficients vector is used to update the signal estimation and the residual, ${\hat{y}}^{\left(t\right)}$ and $r^{\left(t\right)}$. The iteration computes the matrix $P^{\left(t\right)}$ with the projections of the remaining regressors onto the selected one, and the updated matrix of orthogonal basis $Z^{\left(t\right)}$. The procedure is summarized in the Algorithm 1. Regressors are incorporated until the minimum of the BIC criterion is reached. The stopping indicator was defined by the minimum of the BIC written in terms of the normalized mean square error (NMSE) and a penalty term, as in [26],

(8) $$\mathrm{BIC}\left(n_{a}\right)=\mathrm{NMSE}\left(n_{a}\right)+\alpha \;n_{a},$$

where $n_{a}$ is the number of regressors and $\mathrm{NMSE}(n_{a})$ is the NMSE corresponding to this number of regressors.

Three cases of study are analyzed to motivate the upgrading procedure.

### 2.1. Case 1: A Weakly Nonlinear PA

The first case of study is a class AB PA based on Cree’s board for the evaluation of the power GaN HEMT CGH40010, operated at a carrier frequency of 3.6 GHz. Using experimental data acquired for this PA, the procedure of the previous Section is directly applied. The test bench, described in detail in Section 2.3, is here integrated only by the signal generator and the vector signal analyzer. The probing signal was designed with an OFDM format and 15 MHz bandwidth, according to the Long-Term Evolution (LTE) downlink standard. The average power of the input signal was 6 dBm, for which the PA delivers an output average level of 19 dBm. This case with a moderate output level is presented only to illustrate the proposed upgrading procedure in a first approximation. Anyway, the 11 dB of peak-to-average power ratio (PAPR) level produces a peak PA output power of about 30 dBm.
**Algorithm 1:** Upgrading an incomplete model. **Require:**
$X_{a}$, ${\hat{h}}_{a}$, $X_{b}$  1: Initialization: $X\leftarrow [X_{a}\;X_{b}]$, $\tilde{X}\leftarrow X$, $Z^{\left(1\right)}\leftarrow X$  2: **for**
t=1
**to**
*S*
**do**  3:  ${\tilde{X}}_{\left\{t\right\}}\leftarrow 0$  4:  $P^{\left(t\right)}\leftarrow \frac{Z_{\left\{t\right\}}^{(t)H}}{{∥Z_{\left\{t\right\}}^{\left(t\right)}∥}_{2}^{2}}\tilde{X}⊗Z_{\left\{t\right\}}^{\left(t\right)}$  5:  $Z^{\left(t+1\right)}\leftarrow Z^{\left(t\right)}-P^{\left(t\right)}$  6: **end for**
  7: $r^{\left(S\right)}\leftarrow y-X_{a}{\hat{h}}_{a}$, $S^{\left(S\right)}\leftarrow \left\{1,2,\cdots ,S\right\}$  8: $X_{S^{\left(S\right)}}\leftarrow X_{a}$  9: **for**
t=S+1 until stopping criterion is met **do**  10:  $i^{\left(t\right)}\leftarrow \arg\max_{i\notin S^{\left(t-1\right)}}\left|\frac{Z_{\left\{i\right\}}^{(t)H}r^{\left(t-1\right)}}{∥Z_{\left\{i\right\}}^{(t)H}{∥}_{2}}\right|$  11:  $S^{\left(t\right)}\leftarrow S^{\left(t-1\right)}\cup \left\{i^{\left(t\right)}\right\}$  12:  ${\hat{h}}^{\left(t\right)}\leftarrow X_{S^{\left(t\right)}}^{+}\;y$  13:  ${\hat{y}}^{\left(t\right)}\leftarrow X_{S^{\left(t\right)}}{\hat{h}}^{\left(t\right)}$  14  $r^{\left(t\right)}\leftarrow y-{\hat{y}}^{\left(t\right)}$  15:  ${\tilde{X}}_{\left\{i^{\left(t\right)}\right\}}\leftarrow 0$  16:  $P^{\left(t\right)}\leftarrow \frac{Z_{\left\{i^{\left(t\right)}\right\}}^{(t)H}}{{∥Z_{\left\{i^{\left(t\right)}\right\}}^{\left(t\right)}∥}_{2}^{2}}\tilde{X}⊗Z_{\left\{i^{\left(t\right)}\right\}}^{\left(t\right)}$  17:  $Z^{\left(t+1\right)}\leftarrow Z^{\left(t\right)}-P^{\left(t\right)}$  18: **end for**


**Figure 1:**
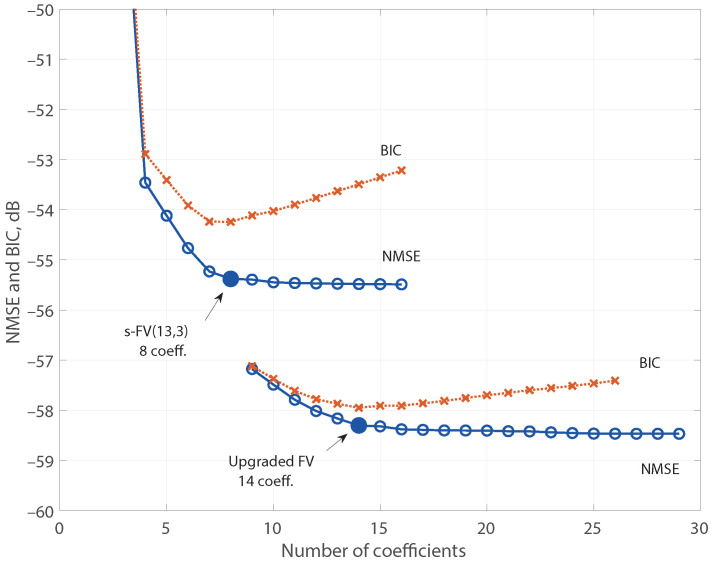
NMSE and BIC vs. number of coefficients for the incomplete s-FV(13,3) and the upgraded models.

**Figure 2:**
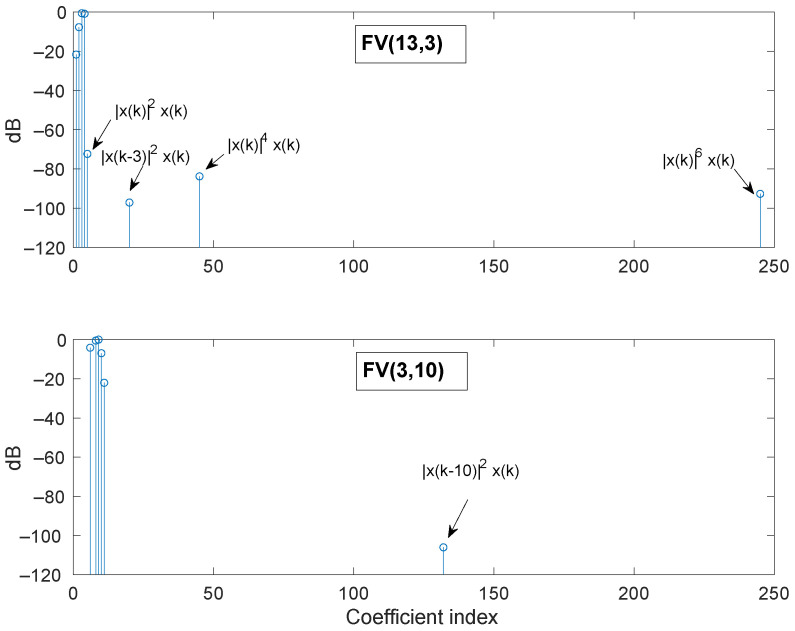
Normalized coefficients. Upper plot: Sub-optimal sparse model identified from the FV(13,3) model. Lower plot: New coefficients of the FV(3,10) structure and incorporated to the upgraded model. Average output level: 19 dBm.

For practical reasons, memory is considered only for FV regressors with nonlinear order below 7. Even in this case, to predict the PA output with an FV model of 13th nonlinear order and a memory of 10 samples, it would be necessary to handle 19,617 regressors, a quantity that exceeds the average computer capabilities. Therefore, we initially considered a model with only three samples of memory length and, therefore, with a shortfall in the regressors stock. The model FV(13,3) contains a raw stock of 248 regressors. The evolution of the NMSE and the BIC as the greedy search includes more active regressors (coefficients) is shown in Figure 1, indicating a model reduced to eight active regressors, denoted as s-FV(13,3), with an NMSE of −55.4 dB. The normalized magnitude of the corresponding estimated coefficients, labeled with the associated regressors, are displayed in the upper plot of Figure 2. Observe that the resulting sparse model is not optimum because of the limited richness of the initial stock of regressors.

**Figure 3:**
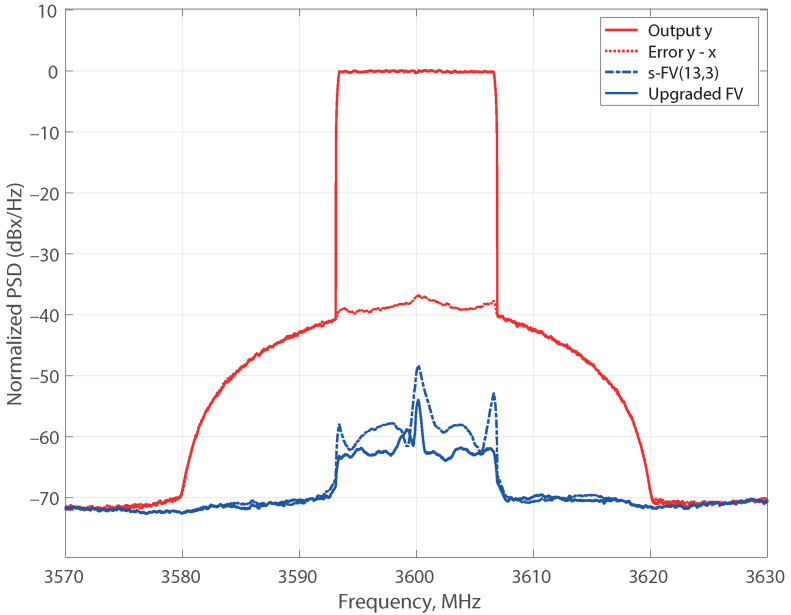
Comparative of the error spectra for the incomplete s-FV(13,3) model and the upgraded FV model (blue traces). Average output level: 19 dBm. All plots have been normalized to make easier comparison with input-output error (red trace).

**Figure 4:**
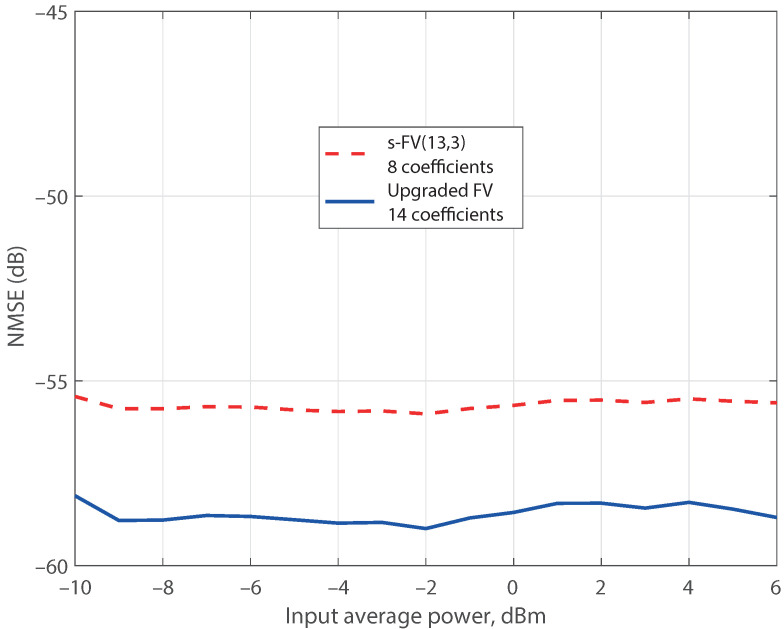
NMSE modeling performance over a 16 dB dynamic range. Coefficients estimated at an average output level of 19 dBm.

As the incomplete set of the s-FV(13,3) model does not embrace regressors with memory larger than three samples, it was updated by incorporating the 737 new regressors of the third nonlinear order FV model with a memory of 10 samples, FV(3,10), and the aforementioned upgrading procedure was applied. The resulting model, denoted as upgraded FV, is also displayed in Figure 1 demonstrating an improved NMSE of −58.3 dB with 14 active regressors. The lower plot of Figure 2 shows the normalized magnitude of the new estimated coefficients, and Figure 3 reveals the comparison of the error spectra for the s-FV(13,3) model and the upgraded FV model (blue traces). The spectrum of the error between x(k) and y(k) is also plotted to have a reference of the distortion generated by the PA. Once the normalized parameters were computed at an input level of 6 dBm, they were straightforwardly scaled to adapt the coefficients to other power levels and the corresponding NMSE were evaluated [26]. In the case of the reduced regressors set derived from the FV(13,3) set (a model with a shortfall in the regressors stock), there are eight active regressors. This pruned model delivers NMSE values below −55 dB in a dynamic range of 16 dB, as it is shown in Figure 4. The pruned model after upgrading contains 14 regressors and the NMSE improves to values of about −58 dB in the complete range. The stable NMSE over 16 dB of dynamic range at the input is an indication of the procedure reliability to select the active regressors successfully.

### 2.2. Case 2: A PA Near Saturation

Nonlinear distortion and memory effects are significantly noticeable in the case of PAs with output levels near saturation, where the efficiency is markedly high. The polynomic behavior of the truncated Volterra series makes the solution diverge near saturation. Hence, an upgrading model approach to overcome this drawback is proposed here.

Taking into account that the Volterra series is linear with respect to the kernels, the *n*th-order Volterra operator

(9) $${\bar{H}}_{n}\left[x\left(k\right)\right]=\sum_{q_{n}=0}^{Q_{n}}h_{n}\left(q\right)\prod_{r=1}^{\frac{n+1}{2}}x\left(k-q_{r}\right)\prod_{r=\frac{n+3}{2}}^{n}x^{*}\left(k-q_{r}\right)$$

can be split into two components

(10) $${\bar{H}}_{n}\left[x\left(k\right)\right]={\bar{H}}_{n}^{\left(a\right)}\left[x\left(k\right)\right]+{\bar{H}}_{n}^{\left(b\right)}\left[x\left(k\right)\right],$$

and the PA output can be expressed as the sum of two Volterra series

(11) $$y_{0}\left(k\right)=\sum_{n=1}^{\infty }{}^{\prime }{\bar{H}}_{n}^{\left(a\right)}\left[x\left(k\right)\right]+\sum_{n=1}^{\infty }{}^{\prime }{\bar{H}}_{n}^{\left(b\right)}\left[x\left(k\right)\right],$$

where the prima indicates that only odd-order terms are included in the sum. As the only assumption is the linearity of the Volterra operators, this result allows the adoption of additional criteria to select each one of the two Volterra series.

Based on the fact that the nonlinear order has not been truncated yet and the Volterra series can be seen as a generalization of the Taylor series, in this subsection, the first part of (11) is chosen as a memoryless function expanded with the Taylor series

(12) $$\phi \left(k\right)=\sum_{n=1}^{\infty }{}^{\prime }{\bar{H}}_{n}^{\left(a\right)}\left[x\left(k\right)\right]=\sum_{n=1}^{\infty }{}^{\prime }c_{n}{\left|x\left(k\right)\right|}^{n-1}x\left(k\right).$$

Observe that the option of a truncated Taylor series would introduce convergence issues. The replacement of the infinite Taylor series by the memoryless function ϕ(k) overcomes this computational instability. Substituting in (11) and truncating the second Volterra series, a similar expression to (1) can be obtained. To make the model (11) unique, it is necessary to adopt a criterion to select the function ϕ(k). Here, a function that maximizes its correlation with y(k) is selected. After collecting the samples of (12) to form the normalized column vector ϕ, the output can be written again as a linear regression

(13) $$y_{0}=a\phi +\sum_{i=1}^{M}h_{i}\xi_{i},$$

where ϕ is a new basis added to the conventional set of Volterra series regressors $\xi_{i}$. Let us remark that ϕ does not have a definite nonlinear order and may not be denoted as a conventional Volterra series regressor, but it has been derived following a Volterra series approach. As this new memoryless basis is derived from the infinite Taylor series (12), it overcomes the inherent instability of a truncated polynomial in the compression region near saturation.

The first step of the proposed procedure is the search for a function presenting the best correlation with the acquired output signal. Once the regressor ϕ is given, it can be considered as the incomplete set of a model lacking active regressors with memory. Then, the supplementary matrix $X_{b}$ is constructed departing from the stock of regressors of a new FV model. Therefore, the updated matrix is

(14) $$X=[\phi \;X_{b}]$$

and the model can be upgraded following the proposed procedure. The analysis is completed experimentally in Section 3 by applying this technique to linearize a PA near saturation.

### 2.3. Case 3: A Generic PA

**Figure 5:**
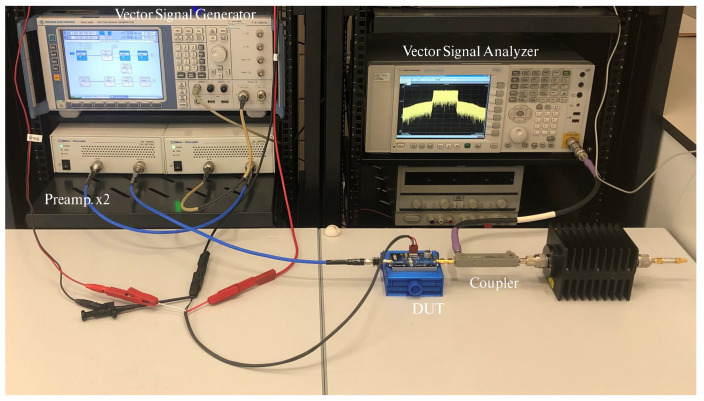
Photograph of the experimental setup.

The model performance was also tested with the class AB PA based on the evaluation board of the Cree’s GaN CGH40010 operated now in a range of output average levels with a maximum of 33.8 dBm and a gain compression of 6 dB. The experimental acquisition was carried out over the test bench whose picture is shown in Figure 5. It was formed of a SMU200A vector signal generator (VSG) from Rohde & Schwarz (Munich, Germany), which was followed by two cascaded Mini-Circuits TVA-4W-422A+ preamplifiers. The output signals were acquired using a PXA- N9030A vector signal analyzer (VSA) from Keysight Technologies (Santa Rosa, CA, USA).

The probe signal was set following a Fifth-generation New Radio (5G-NR) format characterized by a total bandwidth of 30 MHz with 30 kHz subcarrier spacing, 16-QAM symbols over all the subcarriers, a PAPR of 10.5 dB, and a total length of 368,640 samples. A custom Matlab script controlled the settings to modulate the carrier with the 5G-NR waveform in the VSG and acquire samples of the complex envelope of the output signal in the VSA with an oversampling factor of 6, i.e., a sampling frequency of 92.16 MSa/s. Both the DAC converter included in the VSG and the ADC converter included in the VSA presented a resolution of 16 bits. In the VSA, the dynamic range of the measurement was optimized through the equipment settings and by averaging 300 acquisitions of the output signal, thus significantly reducing the noise floor.

**Figure 6:**
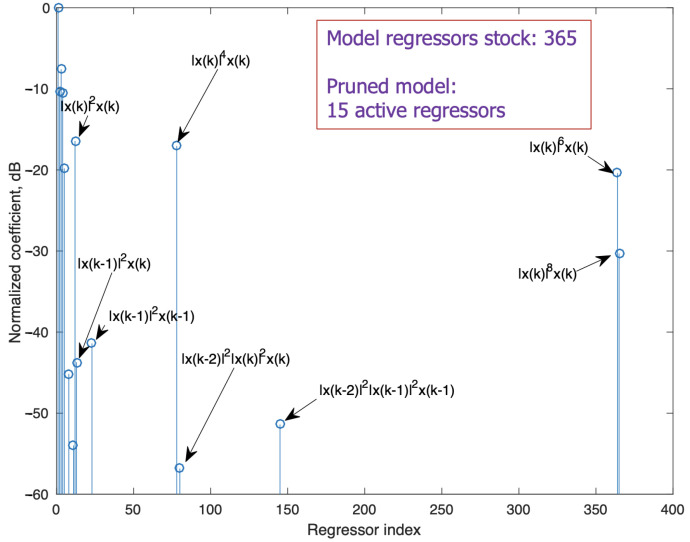
Normalized coefficients. Input signal: 30 MHz 5G-NR. Average output level: 27.4 dBm.

The operation point to obtain the model structure was set to generate an output power level of 27.6 dBm ($P_{i}=-33$ dBm at the input of the two cascaded preamplifiers). To predict the PA output at this level, the model demonstrated in [14] with a ninth nonlinear order and 10 samples of memory length was selected, giving a raw stock of 365 regressors. After a search with the algorithm in [21], the stopping indicator was the minimum of (8) with α=0.14 in the penalty term. The identification procedure, which was performed over 1% of the measured samples, produced an optimum subset composed of only the $n_{a}=15$ active regressors listed in Table 1. The normalized coefficients, estimated with the LS algorithm, are shown in Figure 6 labeled with the corresponding regressors. When validating with the complete length of the signal, this pruned model provides a satisfactory NMSE of −49.2 dB. The two regressors marked with a dagger (†) do not belong to the GMP set of regressors.

**Figure 7:**
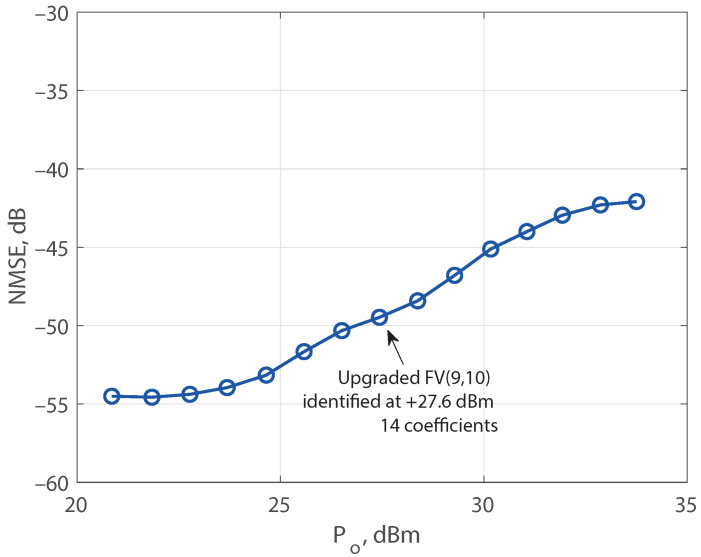
NMSE comparison of the upgraded FV(9,10) model in a dynamic range from 21 to 34 dBm.

To detect if any active regressor is missed, a second search with an FV model was desirable. Given that the search is impractical if the pursuit starts directly with the 19,615 regressors of a ninth nonlinear order FV model with a memory of 10 samples, the upgrading procedure was adopted. First, the search proceeded with the 737 regressors of an insufficient model limited to a nonlinear order of 3 and a memory of 10 samples, FV(3,10), yielding nine identified active regressors. Next, this incomplete model is enhanced with the 246 regressors of a second 9th-order extended model, FV(9,3), and the search procedure was repeated with a result of a pruned model with a total of 14 regressors. Remarkably, 12 of the most significant regressors are the same for both pruned models. The identified structure of 14 active regressors was re-utilized to estimate the model coefficients for output average powers ranging from 21 to 34 dBm. The computed NMSE of the upgraded FV(9,10) model is shown in Figure 7 for the whole dynamic range. The proposed technique for model upgrading was also implemented to obtain the results published in [27].

## 3. Linearization Procedures

### 3.1. Linearization of a Generic PA

The validation of the linearization approach for the generic class AB PA of Section 2.3 was carried out over the aforementioned test bench (Figure 5). As in the previous section, the probe signal was set following a 5G-NR format characterized by a total bandwidth of 30 MHz and a PAPR of 10.5 dB, and the operating point to obtain the model structure was set to an output power level of 27.6 dBm. The attained structure was used for the validation in a signal generator power sweep from −34 to −17 dBm with 1 dB of step size. The output was acquired with a sampling rate of 92.16 MHz, which implies an oversampling factor of 3. This sweep corresponds to an output power range from 21 to 34 dBm and a gain compression varying from 0.6 to 6.0 dB.

**Figure 8:**
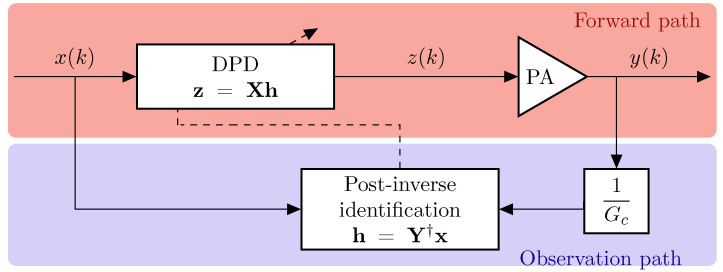
Block diagram of the employed indirect learning architecture DPD.

A standard indirect learning architecture was used for the DPD, as shown in Figure 8, exploiting that the post-distorter function shall be the same as the predistorter [28]. Therefore, the basis functions were chosen to reduce the error between x(k), as the desired output, and the signal $y\left(k\right)/G_{c}$, as the post-distorter input, with $G_{c}$ representing the target gain of the linearized PA. Then, the identified post-inverse model coefficients were copied in the DPD and the predistorted signal z(k) was applied to the input of the PA, thus obtaining a linearized output.

**Figure 9:**
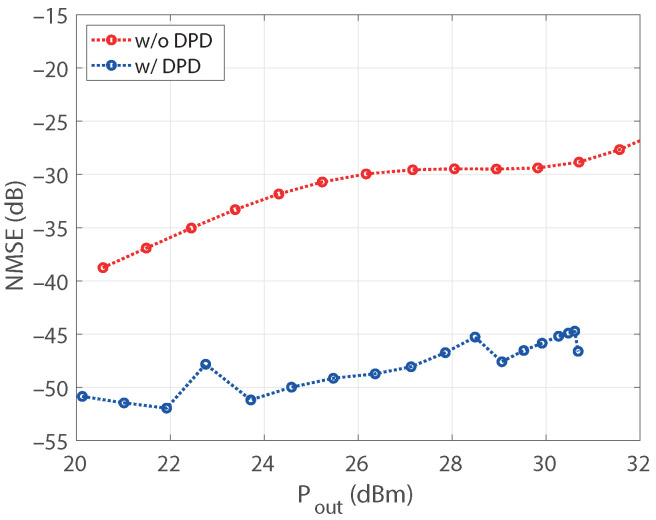
NMSE versus the output power level, without and with the DPD, with a model structure identified at $P_{o}=27.6$ dBm and then reused for all the power levels.

**Figure 10:**
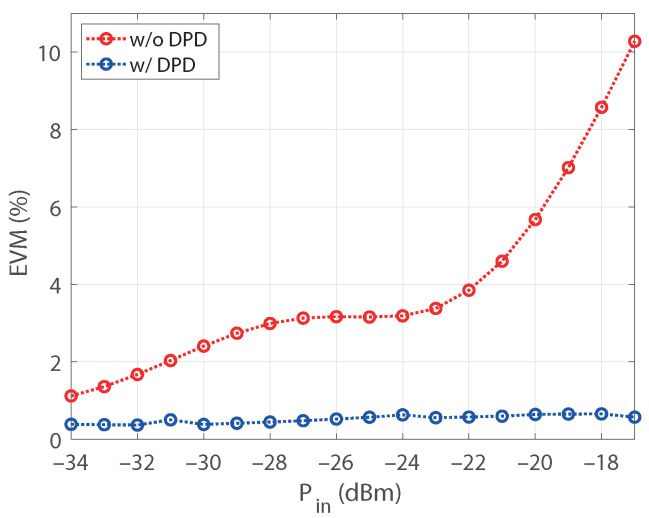
EVM versus the input power level at the VSG, without and with the DPD, with a model structure identified at $P_{o}=27.6$ dBm and then reused for all the power levels.

**Figure 11:**
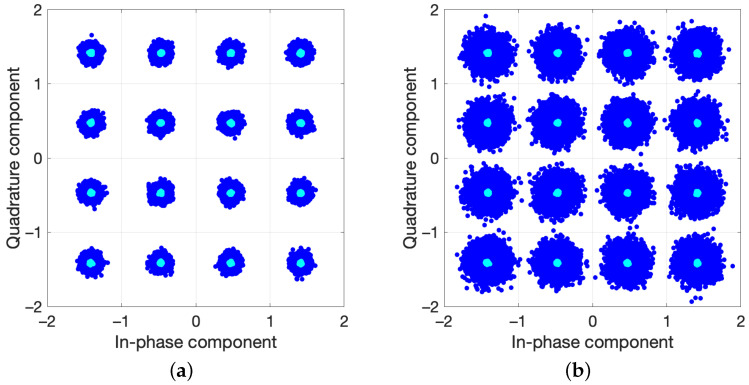
Constellations of the output signal, without (blue) and with DPD (cyan). (**a**) Average output level: 30 dBm, corresponding to an input level of −21 dBm at the VSG (EVM = 4.6% without DPD, and 0.6% with DPD). (**b**) Average output level: 30.7 dBm, corresponding to an average input level of −17 dBm at the VSG (EVM = 10.3% without DPD, and 0.6% with DPD).

**Figure 12:**
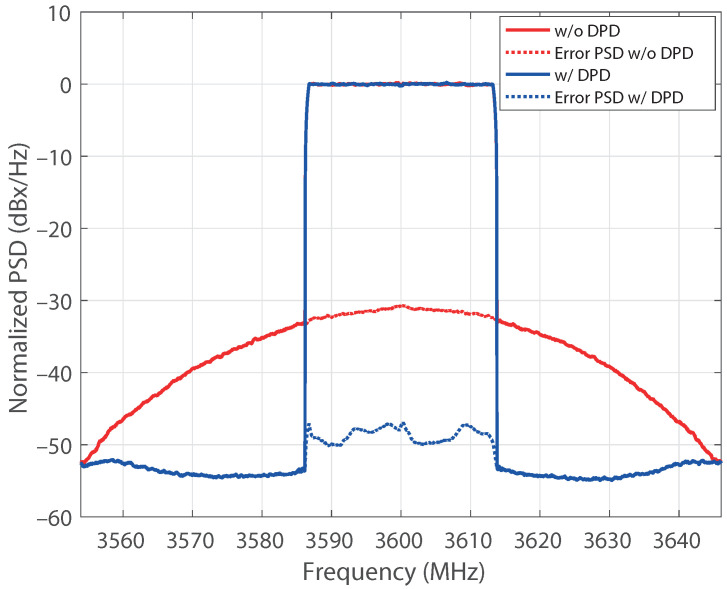
Output spectra without and with DPD. Average output level: 30 dBm.

The upgrading procedure allowed us to evaluate a model with thirteenth nonlinear order and a memory of 10. After pruning the model, the structure exhibited 40 coefficients. Aiming to emphasize the robustness of the results, the active regressors of the structure were identified for the output power of 27.6 dBm taken as a reference. Afterward, the coefficients of a DPD based on that structure were estimated for each power in the sweep. The NMSE of the PA output signal, with and without DPD, is shown in Figure 9 versus the average output power. The NMSE values of the predistorted signal are kept below −45 dB for all the power levels, with an improvement with respect to the nonlinear output of about 15 dB for output levels over 26 dBm. The error vector magnitude (EVM) of the original signal and the linearized one is also provided in Figure 10 versus the input power levels at the VSG. It is worth mentioning that, as the compressed gain $G_{c}$ is considered for the observation path, the designed DPD produces some additional gain reduction with respect to small-signal value [29]. Notice that the target value of EVM with 16-QAM modulation is 12.5%, being the requirement for the in-band nonlinear distortion less demanding. The reduction of the in-band distortion produced by the DPD can also be observed through the constellations for two different input power levels provided in Figure 11. The normalized power spectral density (PSD) of the original signal and for the designed DPD are shown in Figure 12 for an average output power of 30 dBm, where the spectra of the error signals have been also included in dotted lines. The performance of the DPD is clearly illustrated by the reduction produced in the spectral regrowth and the fact that the in-band content in the spectrum of the error signal with DPD is about −48 dB.

**Figure 13:**
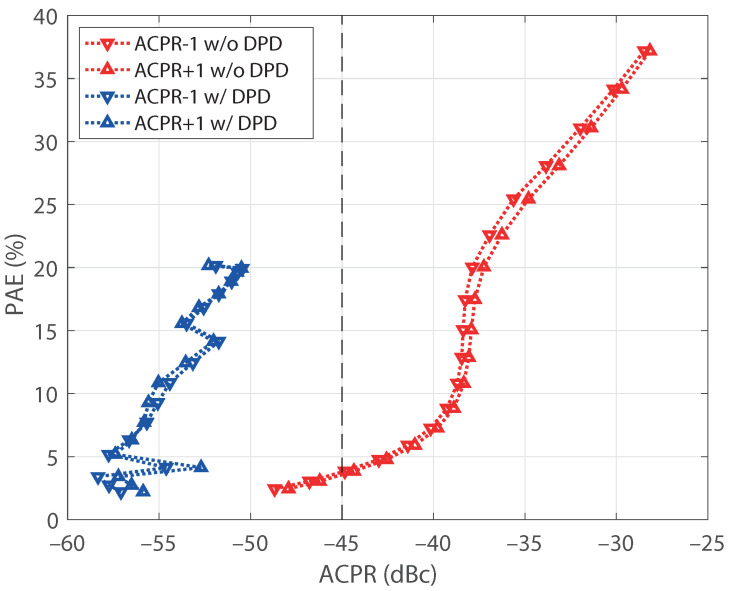
Relationship between PAE and ACPR, without and with DPD.

One of the main advantages of applying a linearization technique to a PA consists in being able to employ it with efficiency values that are unfeasible due to the nonlinear effects constraints. This fact is illustrated in Figure 13, where the power added efficiency (PAE) of the PA is depicted versus its adjacent channel power ratio (ACPR) for both cases, with and without DPD. Note that ACPR values for the measured spectra are calculated as

(15) $$ACPR_{\pm m}\left[\mathrm{dBc}\right]=10\log\frac{P_{\mathrm{side}\;\mathrm{channel}}}{P_{\mathrm{band}}}=10\log\frac{\int_{f_{c}\pm mC-B/2}^{f_{c}\pm mC-B/2}S_{o}\left(f\right)df}{\int_{f_{c}-B/2}^{f_{c}+B/2}S_{o}\left(f\right)df},$$

where $S_{o}\left(f\right)$ is the power spectral density of the output signal, *B* is the integration bandwidth that is equal to the occupied bandwidth of the modulated signal, *C* is the channelization of the wireless standard, and m∈Z represents the considered adjacent channel, being m=+1 for the upper channel and m=−1 for the lower channel.

As expected, the PAE of the PA increases as the output power increases. However, the nonlinear effects of the PAE also produce an increasing ACPR with the output power level. Linearity requirements for mobile communication signals usually demand ACPR values lower than −45 dB to avoid interference in the adjacent channels. For the PA under test, it can be observed that the PAE values are limited below 5% to fulfill this requirement. In contrast, the reduction in the ACPR achieved when a DPD is applied allows us to reach PAE values of up to 20% since all the measured power levels satisfy the ACPR requirement.

### 3.2. Linearization of a PA Near Saturation

The proposed approach was also applied to the linearization of the class J PA operated near saturation. The amplifier under test was designed at 850 MHz over the CGH35015F device, a packaged 15 W GaN HEMT from Cree Inc. The probing signal followed an OFDM format according to the 5G-NR standard, with 20 MHz bandwidth, 30 kHz subcarrier spacing, with 16-QAM symbols over all the subcarriers, 10.2 dB PAPR, and 92.16 MHz sampling frequency. The PA provided an average output power of 33.4 dBm, exhibiting not only gain compression but also gain expansion for the lower levels.

Linearization is focused on cases 2 and 3 since in case 1 the PA was operated in a weakly nonlinear mode. Again, the indirect learning approach was used for the DPD. Following the procedure for case 2 of Section 2, the first step is the search for a static function that maximizes the correlation between the scaled signal $y\left(k\right)/G_{c}$ and x(k). To represent the nonlinear behavior of the inverse system, a complex-valued piecewise polynomial was defined as the static function in this paper, splitting the AM/AM and AM/PM characteristics into five segments and employing a fifth-order polynomial for each of them. The function ϕ(k) allowed assembling a column vector ϕ that was added to the set of Volterra regressors. Then, the identification procedure was employed to find the active set of Volterra regressors departing from an FV model with fifth-order and memory depth of 3. The complete set of Volterra regressors under the FV configuration was 244, while the application of the advanced technique provided a reduced-order model with only 18 coefficients plus the static function.

**Figure 14:**
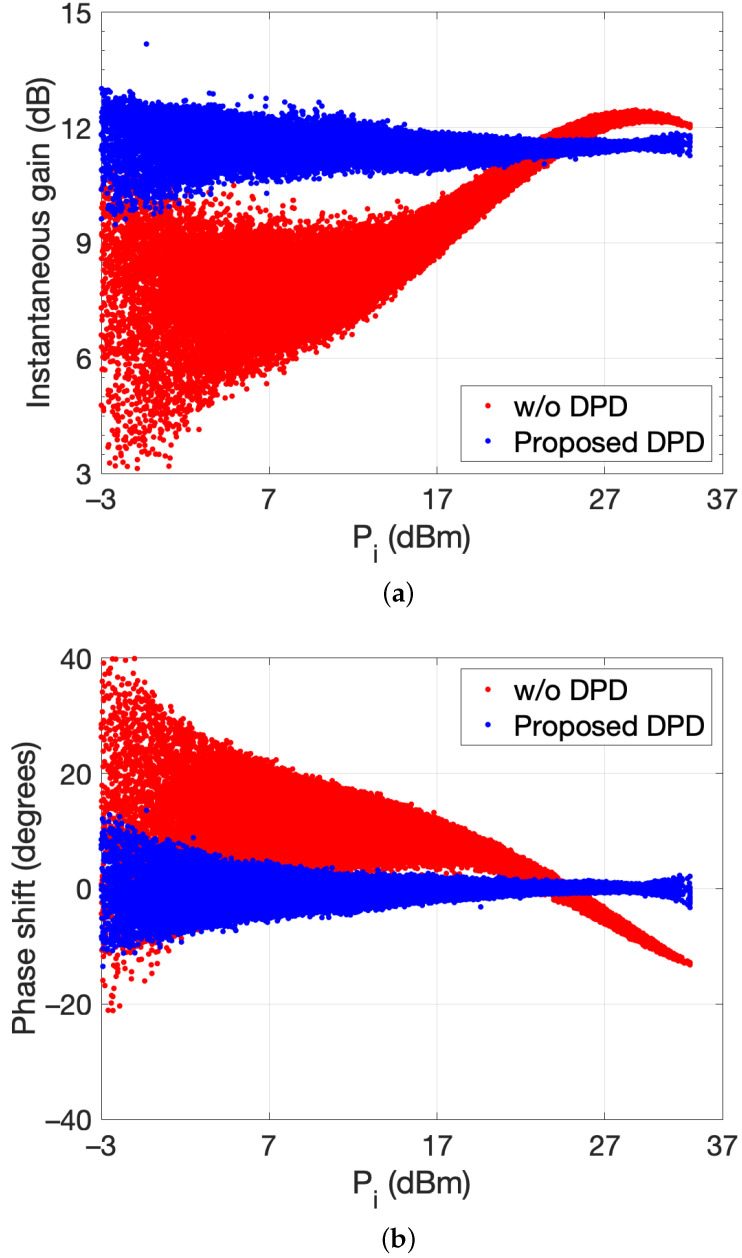
RF dynamic (**a**) gain and (**b**) AM/PM characteristics of the class J PA operated near saturation.

**Figure 15:**
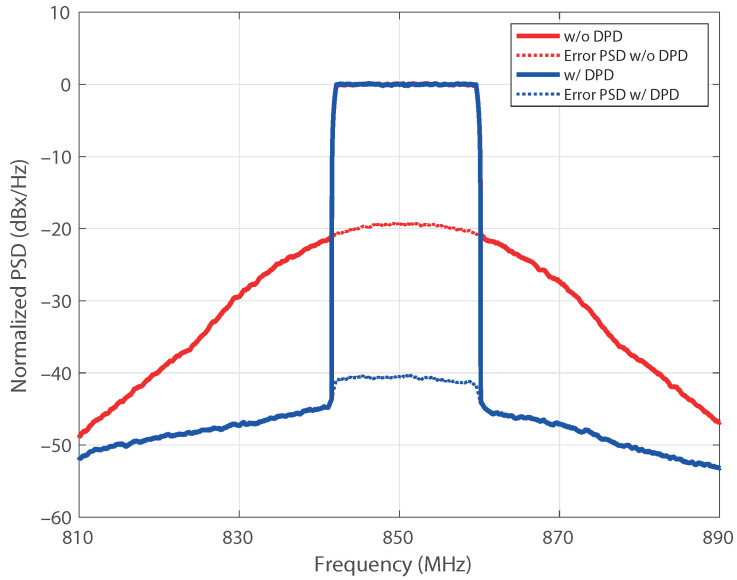
Comparison of the output spectra (solid line) and spectra of the error signals (dotted line) for the class J PA, without and with DPD. Input signal: 20 MHz 5G-NR. Average output level: 33.4 dBm.

Figure 14 shows the RF dynamic gain and the AM/PM characteristics of the class J PA operated near saturation, without and with DPD. Table 2 summarizes the performance of the proposed DPD in terms of the ACPR for the lower and upper channel, the NMSE, and the error vector magnitude (EVM). The present approach provides an improvement over 21 dB of ACPR with respect to the nonlinear output at the same average output level. Figure 15 shows the output power spectra without DPD and when the suggested linearization technique is applied, demonstrating the reduction of the out-of-band emission. The PSD of the error signal between the output of the PA and the scaled input signal illustrates that the in-band error is reduced 40 dB. In-band distortion mitigation can also be assessed in terms of the reduction in EVM from 10.5% to 1.0%, as it is revealed in Table 2. The linearization improvement is also confirmed by the gain attained in the NMSE, which is over 20 dB.

## 4. Conclusions

In this work, a method to upgrade PA models is proposed. The set of active regressors identified for some conventional models (MP, GMP, etc.) can be insufficient to produce an optimal sparse model. On the other hand, sometimes, it is almost impossible to cope with the massive set of FV regressors. An approach to overcome this difficulty by applying an upgrading procedure to deal with the unmanageable set of FV regressors is demonstrated. Focusing on a ninth nonlinear order FV model with a memory of 10 samples, the 19,615 regressors are reduced to 14 active regressors for a generic class AB PA under test after the application of compressed sensing techniques, exhibiting an NMSE of approximately −50 dB. Once the active set of regressors was identified, the corresponding coefficients were estimated in a range of output power levels from 21 to 34 dBm and the model predicts satisfactorily the PA output within a dynamic range of 14 dB. The method was applied to the linearization of the PA by identifying first the DPD structure, i.e., the active DPD regressors, and then the coefficients were estimated for the different power levels. The results indicate a satisfactory NMSE of −45 dB and an ACPR below −50 dB in both adjacent channels at all levels of the dynamic range.

It has been demonstrated that a memoryless static function can be used as a legitimate regressor in a non-truncated Volterra model, and this regressor can be also upgraded following the exposed procedure. This approach was applied to the linearization of a class J PA operating in conditions near saturation. The proposed method has been experimentally validated with the amplifier driven by a 20-MHz 5G-NR signal. After linearization with the proposed DPD, the results show more than 21 dB of ACPR and NMSE improvement with a reduction of the EVM from 10.5% to 1.0%.

## Figures and Tables

**Table 1:** Identified regressors. Input signal: 30 MHz 5G-NR and $P_{\mathrm{out}}=27.6$ dBm.

Regressor	Normalized Coefficients (dB)
x(k)	0
x(k−1)	−10.29
x(k−2)	−7.60
x(k−3)	−10.45
x(k−4)	−19.82
x(k−7)	−45.17
x(k−10)	−53.97
${\left|x\left(k\right)\right|}^{2}x\left(k\right)$	−16.43
${\left|x\left(k-1\right)\right|}^{2}x\left(k\right)$	−43.71
${\left|x\left(k-1\right)\right|}^{2}x\left(k-1\right)$	−41.39
${\left|x\left(k\right)\right|}^{4}x\left(k\right)$	−17.05
${\left|x\left(k-2\right)\right|}^{2}{\left|x\left(k\right)\right|}^{2}x{\left(k\right)}^{†}$	−56.83
${\left|x\left(k-2\right)\right|}^{2}{\left|x\left(k-1\right)\right|}^{2}x{\left(k-1\right)}^{†}$	−51.40
${\left|x\left(k\right)\right|}^{6}x\left(k\right)$	−20.39
${\left|x\left(k\right)\right|}^{8}x\left(k\right)$	−30.31

**Table 2:** Linearization performance of the proposed DPD technique for the PA near saturation. $P_{\mathrm{out}}=+33.4$ dBm, 5G-NR 20 MHz.

Case	ACPR (dBc)		NMSE (dB)	EVM (%)
	Lower Channel	Upper Channel		
Without DPD	−26.8	−26.6	−17.9	10.5
Proposed DPD	−47.7	−48.8	−39.1	1.0

## Data Availability

The data presented in this study are available on reasonable request from the corresponding author.
